# Study on the development and integration of 3D‐printed optics in small‐scale productions of single‐use cultivation vessels

**DOI:** 10.1002/elsc.202100131

**Published:** 2022-03-18

**Authors:** Louis Maximilian Kuhnke, Johanna Sophie Rehfeld, Christian Ude, Sascha Beutel

**Affiliations:** ^1^ Institute of Technical Chemistry Gottfried‐Wilhelm‐Leibniz Universität Hannover Hannover Germany

**Keywords:** 3D‐printing, deepwell, individual labware, optical modification

## Abstract

Integrating optical sensors and 3D‐printed optics into single‐use (SU) cultivation vessels for customized, tailor‐made equipment could be a next big step in the bioreactor and screening platform development enabling online bioprocess monitoring. Many different parameters such as pH, oxygen, carbon dioxide and optical density (OD) can be monitored more easily using online measuring instruments compared to offline sampling. Space‐saving integrated sensors in combination with adapted optics such as prisms open up vastly new possibilities to precisely guide light through SU vessels. This study examines how optical prisms can be 3D‐printed with a 3D‐inkjet printer, modified and then evaluated in a custom made optical bench. The prisms are coated or bonded with thin cover glasses. For the examination of reflectance performance and conformity prisms are compared on the basis of measured characteristics of a conventional glass prism. In addition, the most efficient and reproducible prism geometry and modification technique is applied to a customized 3D‐printed cultivation vessel. The vessel is evaluated on a commercial sensor‐platform, a shake flask reader (SFR) vario, to investigate its sensing‐characteristics while monitoring scattered light with the turbidity standard formazine and a cell suspension of *Saccharomyces cerevisiae* as model organism. It is demonstrated that 3D‐printed prisms can be used in combination with commercial scattered light sensor‐platforms to determine OD of a microbial culture and that a 3D‐printed unibody design with integrated optics in a cultivation vessel is feasible. In the range of OD_600_ 0–1.16 rel.AU a linear correlation between sensor amplitude and offline determined OD can be achieved. Thus, enabling for the first time a measurement of low cell densities with the SFR vario platform. Moreover, sensitivity is also at least three times higher compared to the commonly used method.

**Abbreviations**
CAD: computer aided design
OD: optical density
OMW: optically modified well
SFR: shake flask reader
SQ: status quo
SU: single use

## 1 INTRODUCTION

Nowadays, 3D‐printing technology offers a fast and cost‐effective way to produce customized and tailor‐made components from plastics [1]. In most cases, these are novel components that are difficult or impossible to produce using conventional methods [2]. Since 3D‐printed parts usually have a very specific function, it is difficult to compare them with conventionally produced parts [3]. Over time, 3D‐printing technology has evolved to allow the printing of many other materials, for example, silicone [4], metals and  even ceramics [5, 6]. In the area of bioprocess technology, additive manufacturing allows the production of new lab devices [7, 8], in addition to individual prototype applications for existing labware [9].

This paper is focused on investigating the use of 3D‐printing to produce a SU deepwell (DW) single piece, in which the well is optically functionalized. In this optically modified well (OMW), the concentration of a particle or cell suspension can be measured enabling biomass monitoring. The objective of optical functionalization is to improve key specifications of scattered light sensors in a turbulent oscillating flow regime (shaken operational mode) like sensitivity, insensitivity to perturbations and increasing degree of freedom in terms of measuring location.

In the current measurement method, or status quo (SQ), the light enters the vessel vertically from below through the bottom of the cultivation vessel [10]. In this study, by the use of prisms, the light is deflected by 90° and deflected laterally, and the scattered light measurement takes place in the horizontal plane. Thus, light reflections of the vessel cap or random reflections at the liquid‐air interface should affect measurements much less, which is especially important for measuring low optical densities (OD_600_ < 1.0 rel.AU), for example, in the lag phase of microbial cultures or in mammalian cell suspensions like Chinese Hamster Ovary (CHO) [11]. The prisms that deflect the light at 90° into the OMW are modified in different ways and compared with a commercial prism made of glass, since a comparison allows a quick assessment of the optical quality of these prisms.

For this purpose, an optical bench is constructed using a mixed setup of high precision linear translation stages and FDM‐printing in order to compare the prisms. A particular challenge with 3D‐printed optics is the transparency of the printed optical components [12]. The resolution in the z‐dimension influences the optical properties due to steps and usually only a coating improves the optical properties and smoothens the surface [13]. In terms of printing deflection prisms, it is crucial to prevent material steps and artifacts at the hypotenuse which suppresses total reflection of light and leads to light loss by dispersion instead.

As for this work a 3D‐inkjet printer is used, which has a print head with a predefined trajectory. Therefore, it was also important to investigate the influence of the CAD‐model orientation on the optical properties and consequently how parts should be placed on the plate [14]. Since deflection prisms have flat surfaces, one solution is to bond cover glasses to improve the reflective properties of the hypotenuse. This also means that it involves an increased effort, but a lower effort than other optimization processes such as polishing or coating of the surfaces [13, 15]. The research on printing of vessel integrated optics also showed, that printing orientation and relative tilt of optics toward each other is crucial, since 3D‐printers generate an orientation specific surface pattern of different quality [16]. The automatic integration of support material, was also a challenge since the contact faces of support material and construction materials exhibit poor optical quality [17].

Due to the growing diversity of 3D‐printing methods and different commercial printer types as well as different post processing techniques to improve surface quality, the following work is dedicated to investigate a suitable combination of those two factors in order to reach for one piece cultivation vessels with functional optics to guide light for different sensing applications.

**PRACTICAL APPLICATION**

By using 3D‐printing technologies a single use cultivation vessel with integrated optics is designed and printed in one piece to determine optical density (OD) of a microbial culture enabling biomass monitoring. Integrated prisms which deflect the light by 90° facilitate the scattered light measurement in the horizontal plane rather than in the common method where the light enters the vessel vertically from below. The vessel is evaluated on a commercial sensor platform. It is shown that at low optical densities (OD_600_ < 1.0 rel.AU) a linear correlation between sensor amplitude and offline determined OD is achieved. Compared to the commonly used method, sensitivity of the new vessel is on average at least three times higher. The presented vessel could pave the way to enable noninvasive biomass monitoring of mammalian cell cultivations with generally low optical densities.

## 2 MATERIALS AND METHODS

### 2.1 Optical bench overview

**FIGURE 1:**
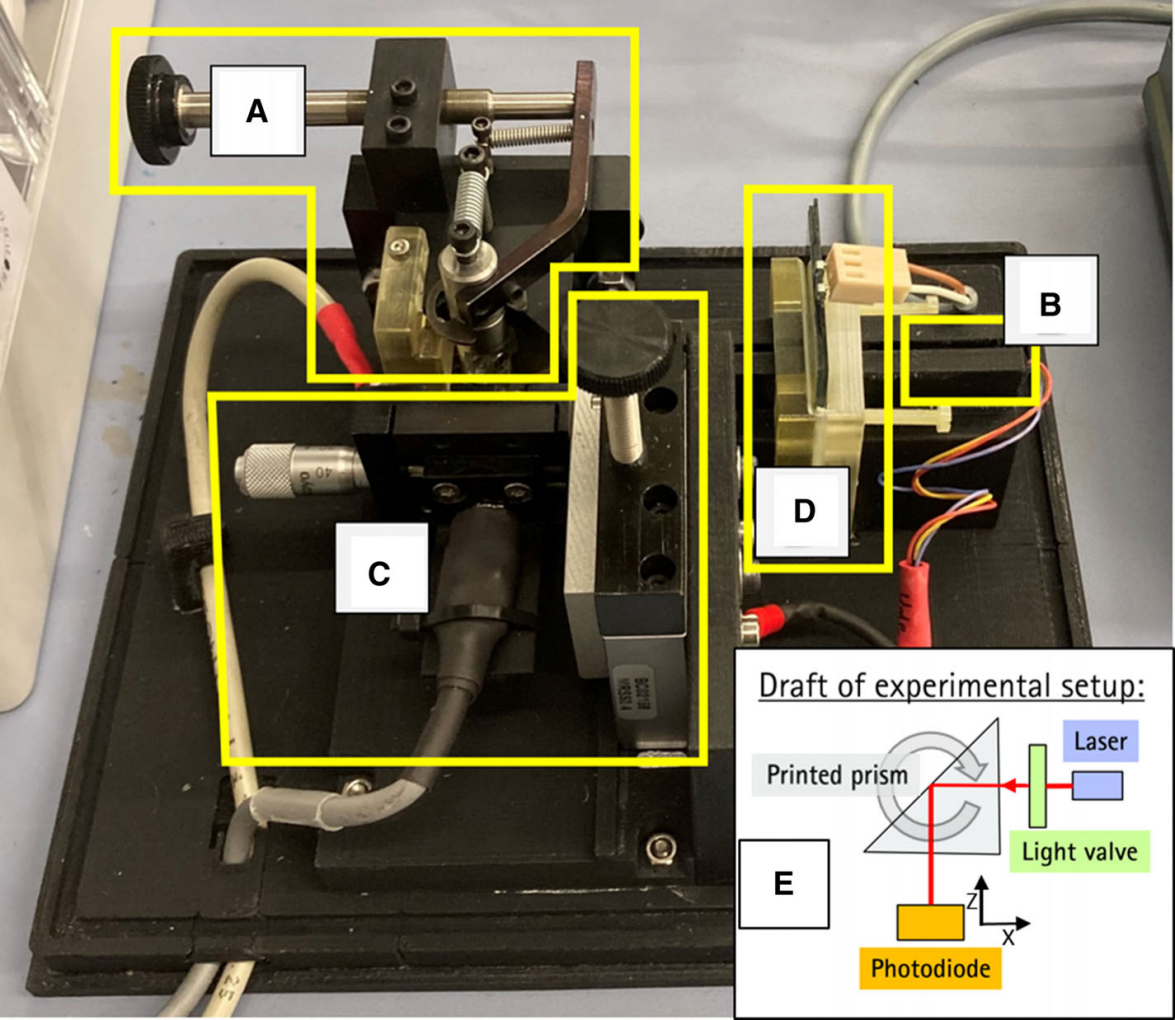
The optical bench used for prism evaluation. (A) is an adjustment unit in which the prisms are installed and can be aligned. There is the first photodiode located; (B) is the holder in which the laser is placed; (C) is the second photodiode, which is attached to a double‐tube linear unit, which in turn is attached to another 90° rotated and tilted double‐tube linear unit. This second photodiode picks up the signals for the measurements; (D) is the light valve; (E) Draft to further clarify the experimental setup. The cover is not shown

A custom‐designed 3D‐printed optical bench is used to evaluate the prisms. This optical bench is shown in Figure 1. All parts and components in this work are designed using the CAD software “Autodesk Inventor Professional 2020” (Autodesk, Inc., SanRafael, USA). The optical bench consists of a holder for a laser and a holder for a light valve that changes the amplitude of the laser, an adjustment unit for the prisms with a photodiode, and an adjustment unit for another photodiode that converts the redirected light into an electrical signal. In addition, there is a cover so that no ambient light can influence the measurement. To further reduce randomly scattered light, prisms near structures and the interior of the cover were coated with black, light‐absorbing paint Black 3.0 (Culture Hustle, UK).

### 2.2 Components

The backbone of the optical bench consists of 11 individual parts that were glued together with plastic adhesive. The cover, the basic structures and mounts for the laser and the adjustment units for the sample prism as well as the mount for the adjustment unit of a photodiode were printed with a fused deposition modeling (FDM) printer MakerBot Replicator Z18 (MakerBot Industries LLC, USA). The material used is a black matte polylactid acid (PLA) filament (DAS FILAMENT Inh. Roman Stieben, Germany). The mount for the Light Valve 3627 (Adafruit Industries LLC, USA) was printed with an Agilista‐3200W (Keyence Corporation, Osaka, Japan) using AR‐M2 (Keyence Corporation, Osaka, Japan) as material.

A Right Angle Prism (#35‐900, Edmund Optics Inc., Barringtin, USA) made of silica glass is used as reference prism. The three‐sided prism with the base of an isosceles right triangle is used for 90° deflection. The prism is mounted on an axial rotatable adjustment unit, which can be used to adjust the entrance angle of the laser beam to 90° (modulated red dot laser module MI650‐1‐5, Picotronic GmbH, Koblenz, Germany) with a beam diameter of 1 mm to one of the catheti. Directly behind the hypotenuse of the prism is a photodiode Model S5971 (Hamamatsu Photonics K.K, Hamamatsu, Japan) with a photosensitive area size of 1.1 mm^2^, which detects the transmitted or scattered light (light loss that is not reflected at the hypotenuse) and thus collects the data for the measurements.

The main photodiode S5971 is mounted in a ZX‐adjustment unit consisting of two double‐tube linear units angled 90° to each other. It is intended to collect the 90°‐deflected light from a prism sample and is positioned 17 mm away from the prism. To further reduce the intensity of the laser module a small piece of biaxially oriented polyethylene terephthalate (BoPET) film (MFD‐2HD, Sony, Tokyo, Japan) is placed in front of the photodiode.

The signals of the photodiodes are recorded by the oscilloscope of the commercially available STEMlab Red Pitaya board. The light valve is driven by a JOY‐IT JDS2915 (SIMAC Electronics, Neukirchen‐Vluyn, Germany) signal generator (settings: 5.6 V amplitude, 100 Hz, offset  =  0, duty  =  50%).

### 2.3 Printed prisms

**FIGURE 2:**
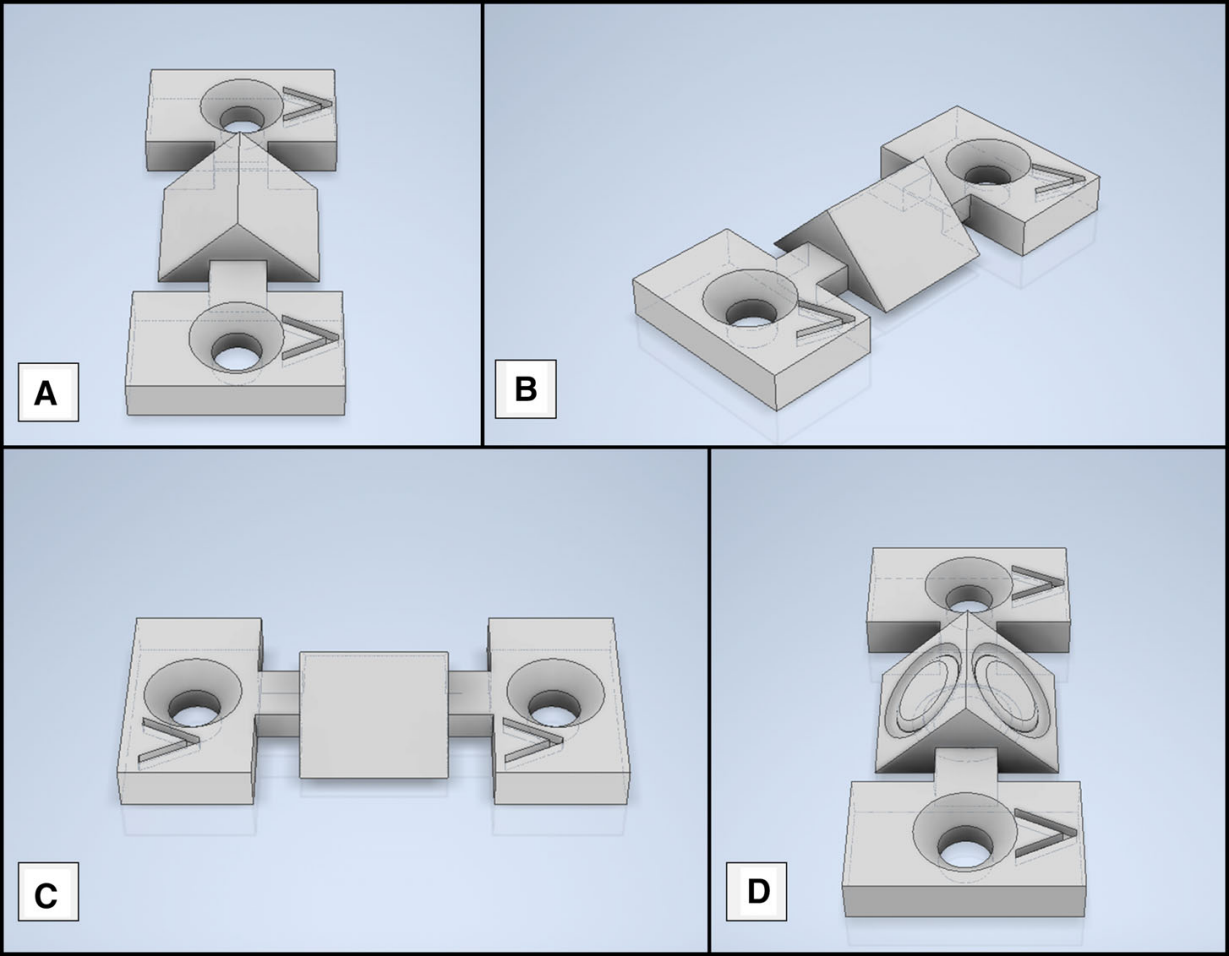
CAD‐models of the printed prisms used. The embedded arrows point in the direction of travel of the print head. (A) Prism in the 0° orientation; (B) Prism in the 45° orientation; (C) Prism in the 90° orientation; (D) Prism with an integrated drip edge (DE)

The 3D‐printed prisms to be examined have similar dimensions to the glass prism (base area: 5.0 mm × 7.1 mm). In addition, they have two bars on the sides, which connect them with two mountings for screws. In Figure 2, CAD‐models of the printed prisms used are shown.

The AR‐M2 material is printed with the inkjet 3D‐printer Agilista 3200W using the proprietary software Modeling Studio (Keyence Corporation) as slicer. During the printing process, the hypotenuse of the printed prisms is always on the printing plate. Regarding the print settings, a glossy printing mode is selected so that the prisms are not coated with a layer of support material.

The influence of the alignment of the prisms to the direction of travel of the print head is analyzed. Hence, the prisms are arranged on the virtual print bed with the surfaces of the catheti pointing in the movement direction of the print head and, depending on the input, are rotated orthogonally to the base surface.

Examined are prisms with the rotation angles 0°, 45° and 90°. In Table 1 six variations of prisms investigated for this paper are shown. The first printing batch contained three replicates of each specimen. For the second batch six replicates of each specimen were printed. The used lacquer luxaprint shellac (DETAX, Ettlingen, Germany) is always applied by hand with a brush for all specimens.

**TABLE 1:** Abbreviations and processing methods of the printed prisms

Specimen abbreviation	Processing methods
–	Unmodified, only cleaned up with ethanol.
C (coated)	Specimens cleaned and coated with the transparent UV‐curing acrylic lacquer luxaprint shellac (DETAX, Ettlingen, Germany).
3GCS (three glass cover slips)	Cleaned and bonded with three round cover glasses (Ø 4 mm (1. print batch) and Ø 5 mm (2. print batch), thickness 0.14 mm) for microscopes on each surface using the same acrylic lacquer luxaprint shellac.
2GCS (two glass cover slips)	Cleaned and bonded with round cover glasses (Ø 4 mm, thickness 0.14 mm) for microscopes on the hypotenuse and cathetus which the laser enters using the same acrylic lacquer luxaprint shellac. All other sides are not modified.
DA (different angles)	Prisms arranged and printed at different rotation angles (20°, 30°, 40°, 50°, 80°, 110°) on the printing plate, chosen by sight for smoothest surface, also cleaned and coated with the acrylic lacquer luxaprint shellac.
DE (drip edge)	A set of prisms (only 0° orientation) with a new geometry with a lowered round surface with a cavity to serve as a drip edge and distribute the acrylic lacquer luxaprint shellac more evenly.

### 2.4 Measuring method

To achieve consistent conditions for the measurements, the distances from light valve to prism and photodiode to prism are the same for all measurements. One measurement ran for 2.2 s and approximately 16,000 readings were recorded during this time using a Python script (Build Version 2.7.9). In addition, measurements were made according to the following protocol:

- The Red Pitaya board and signal generator were turned on and ran for at least 2 h to get up to operating temperature.
- Interference signals are reduced by grounding the Red Pitaya and the adjustment unit.
- Since the photodiode, which receives the deflected light, cannot be moved in the direct proximity of prism to be measured, the prism and the photodiode are adjusted with the adjustment units in that manner that the highest signal is achieved at the oscilloscope of the Red Pitaya board (ZX peak signal).
- For every new series of measurements, the glass prism in the optical bench is remeasured and used as a reference prism.
- Using a Python script to record data from the oscilloscope, the light valve is switched on simultaneously.

### 2.5 Random error due to installation and removal

To determine the random error caused by installing and removing a prism, the glass prism is installed and removed six times and measured on the optical bench. Since the standard deviation of the recorded readings is less than 1% of the measured average value, no further adjustments were made to the measurement protocol.

### 2.6 Data used and data analysis

The light, which is deflected by 90° and reaches the photodiode, is converted into a voltage value by a transimpedance amplifier (TIA) and digitalized by an analog‐to‐digital converter (ADC) in the electronics. To receive a comparison value of the signal amplitude all voltage values are averaged and the values of the respective printed prisms are normalized to the values of the reference prism by the Equation (1):

(1) $$\begin{array}{ccc} & & \mathrm{Amplitude}\;\mathrm{Characteristic}\;\mathrm{Number}\left(\mathrm{ACN}\right)\\ & & \;=\frac{\mathrm{Average}\;\mathrm{Value}\;\mathrm{Printed}\;\mathrm{Modified}\;\mathrm{Prism}\left[V\right]}{\mathrm{Average}\;\mathrm{Value}\;\mathrm{Reference}\;\mathrm{Glass}\;\mathrm{Prism}\left[V\right]}. \end{array}$$

Thus, the higher the amplitude characteristic number (ACN), the better the property to redirect the light by 90°.

### 2.7 Design of the OMW

**FIGURE 3:**
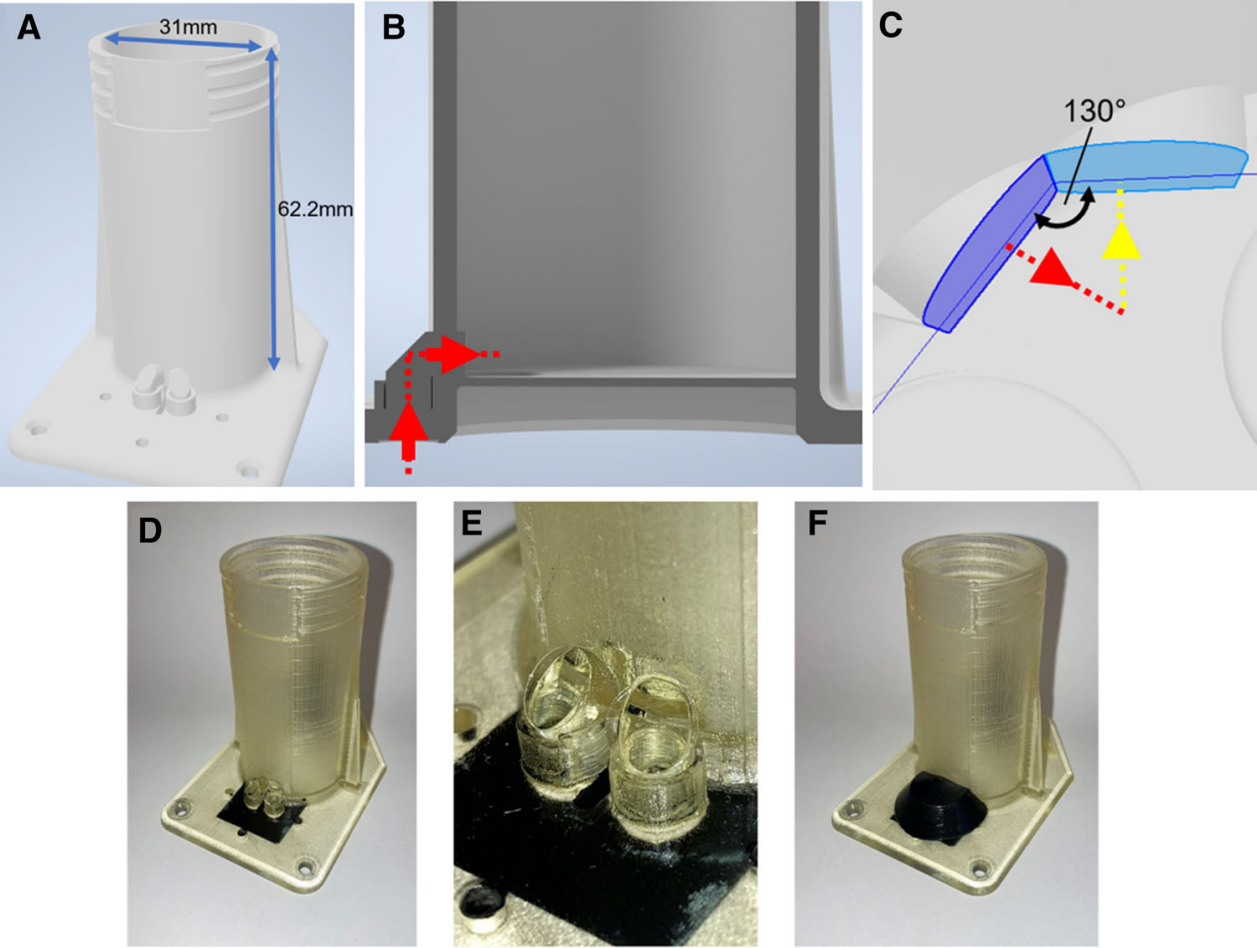
CAD‐model of the OMW with used dimensions. (A) model as a whole; (B) a cross section of the CAD‐model of one light guide which also depicts the intended beam path for the excitation light (red dashed line and pointers); (C) close‐up view from above of the inside of the well showing the alignment of the catheti of the two prisms used and the intended beam path for the scattered light measurement (excitation light path in red, detection light path in yellow). The printed OMW with blackened bottom: (D) OMW, the holes on the base of the vessel are meant for screws to mount it on the SFR vario; (E) close‐up view of the hypotenuses of the prisms which are already bonded with cover glasses; (F) OMW with the blackened prism cover attached

The base and bottom surfaces of the wells are based on the dimensions of a round well of a tissue culture 6 wellplate, Standard, F 83.3920 (Sarstedt, Nümbrecht, Germany). The CAD‐model and the corresponding dimensions are shown in Figure 3. The prism‐added optical guide of the OMW passes from the outside through the wall of the OMW and meets inside the well, with the exit panes of light guides (excitation and detection) angled at 130°. The glossy printing mode is used. Figure 3D shows the printed vessel. The holes in the corners of the base of the vessel are used to mount it on a shake flask reader (SFR) vario by screws. The surfaces of the prisms are each bonded with cover glasses outside the well (see Figure 3E). Since it is difficult to reach the surfaces inside the well, they were only coated with the acrylic lacquer. To prevent ambient light from affecting the measurement results, the surrounding area of the prisms is painted black on the bottom side and the hypotenuses of the prisms are covered with a blackened cap to shield it from mechanical damage (see Figure 3F).

### 2.8 Optical sensor for scattered light measurement

In this work the SFR vario from PreSens Precision Sensing GmbH (Regensburg, Germany) was used. The SFR vario is a multisensory platform for online monitoring of pH, dissolved oxygen and biomass in shake flask. The biomass measurement is based on scattered light detection using a LED and a photodiode. The amplitude of the scattered light is then recorded by the SFR vario.

### 2.9 Preparation of solution for calibration procedure

The calibration was carried out with the turbidity standard formazine (ISO 7072) and a cell suspension of *Saccharomyces cerevisiae (S. cerevisiae*, strain NCYC 1024). The cell suspension was produced by cultivation in complex YPD media (10 g/L yeast extract (Carl Roth), 20 g/L peptone (Sigma‐Aldrich), 10 g/L glucose (Carl Roth)) for 24 h. The cells were harvested by centrifugation at 4.000 × *g* for 15 min and 4°C and at early stationary phase at an OD_600_ of 24 rel.AU. For the offline determination of OD_600_ a photometer (Libra S80, Biochrom GmbH, Cambridge, UK) was used. OD was measured at 600 nm in a d  =  1.00 cm cuvette (Sarstedt AG &Co. KG). The supernatant was discarded and the resulting cell pellet was resuspended in 0.9% (w/v) NaCl‐solution (Carl Roth) out of which sequential dilutions for the calibration procedure were produced. Formazine dilutions were created with deionized water (Arium 661 Ultrapure water system, Sartorius Stedim Biotech S. A, Göttingen, Germany).

Calibrations were performed by measuring the scattered light signal online, using the SFR vario and determination of OD_600_ offline. The data were used to calibrate scattered light in dependency of OD_600_.

### 2.10 Online versus offline measuring calibration procedure

For the online determination of the 180°‐scattered light signal the OMW was setup on the SFR vario, which was mounted on an orbital shaker with 25 mm shaking diameter (Solaris2000, ThermoFisher Scientific, Waltham, USA). The well was filled with 12.5 mL calibration solution (formazine or cell suspension), which corresponds to 20% of the total well volume. The shaking frequency was 300 rpm with the trigger angle of 180° and an illumination time of 68.9 ms. For each calibration point 10 data points were recorded.

### 2.11 Evaluation of used optics in the OMW

To evaluate the OMW, calibrations of the scattered light signal in dependency of offline OD_600_ were performed, where the light is redirected into the well through the prisms. The calibration using OMW was compared with the calibration of factory default method, the SQ method, where the light is directly directed vertically into the well through the bottom. Calibrations were performed first with the turbidity standard formazine and second with a cell suspension of *S. cerevisiae*. Since the scattered light measurement via the prisms is expected to lead to a significant improvement in the low optical density (OD) range, a calibration range of OD_600_ 0–5.5 rel.AU was specified. The root mean squared error of prediction (RMSEP) was calculated using Equation (2)

(2) $$RMSEP=\sqrt{\frac{\sum_{i=1}^{n}{\left(f{\left(x\right)}_{i}-y_{i}\right)}^{2}}{n}},$$

where $f{\left(x\right)}_{i}$ is the value predicted by the calibration function, $y_{i}$ is the reference value and n is the number of predicted samples. The RMSEP is an estimator, that estimates the error, that is, accuracy [18], of the calibration modell used for reference value calculation from sensor signal (OD).

The limit of detection (LoD) is the lowest concentration which can be reliably measured and the limit of quantification (LoQ) is the lowest concentration which can be quantified [19]. Based on the guidelines of the International Conference of Harmonisation (ICH) the LoD is calculated with Equations (3) and (4)

(3) $$LoD\;=\;\frac{3.3\;\cdot \;\sigma }{S},$$

(4) $$LoQ\;=\;\frac{10\;\cdot \;\sigma }{S},$$

where σ is the standard deviation of the blank sample, which is the medium without cells and S which is the slope of the calibration curve [20].

## 3 RESULTS

### 3.1 Results of prism testing

**FIGURE 4:**
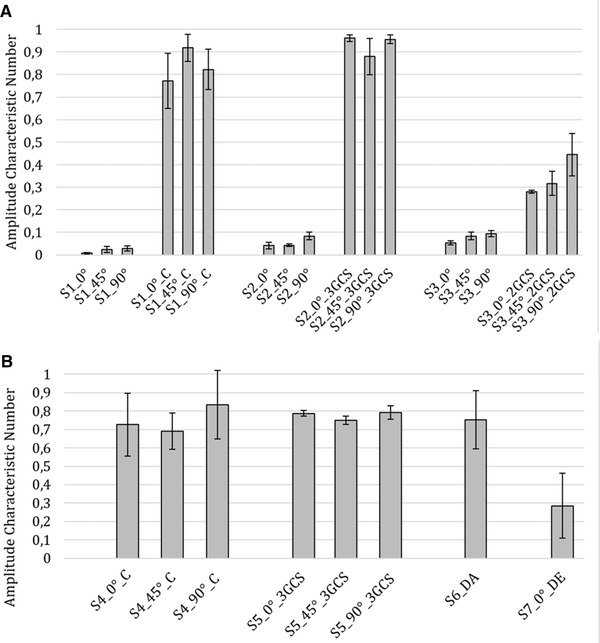
Measured Amplitude Characteristic Number (ACN) values plus Standard Deviation (SD) (error bars). (A) Different specimens (S1‐S3) in different printing directions (0°, 45°, 90°), either coated (C) with lacquer, bonded with 3x glass coverslips (3GCS) or bonded with two glass coverslips (2GCS). All prisms originated from one printing batch (batch 1, three replicates for each specimen). (B) Measured amplitudes plus standard deviation. Specimens (S4‐S7) in different printing directions (0°, 45°, 90°), which were either coated (C), bonded with three glass coverslips (3GCS), printed at different angles (DA, 20°, 30°, 40°, 50°, 80°, 110°) or have a drip edge (DE). All prisms originated from one print batch (batch 2, six replicates for each specimen)

The goal for the printed prisms was to achieve the highest possible reflection at the hypotenuse surface and lowest scattering by the cathetus surfaces. The measurement results are intended to have a low standard deviation. It is predictable that the glass prism and the prisms with the bonded cover glasses give the best results, as can be seen in Figure 4A. In a first experiment it was determined that the rough unmodified surfaces of the cleaned prisms strongly scatter the laser light significantly. For better comparability, the specimens of the prisms from the respective print batch are shown in one diagram.

As expected, the unprocessed prisms S1, S2 and S3 of any printing direction do not reach ACNs compared to the processed prisms. The standard deviation (SD) of the prisms S2 with three bonded cover glasses (3GCS) is lower, because the smooth surface of the machine‐made cover glasses has a high influence on the redirection of the light and scatters the light less. For the S3 variant with only two bonded cover glasses, an ACN of 0.28–0.45 was measured depending on the printing orientation, which probably means that even a single unprocessed surface of a prism strongly scatters the light instead of reflecting it. Since the SD is unusually high for the S2_45°_3GCS specimen, the experiment is repeated with the coated and three‐bonded variant, since it achieved the best results. This time six instead of three replicates of each specimen were examined.

In Figure 4B, these results are shown next to the results for prisms at different angles and for prisms with a drip edge (DE). This time, the unprocessed specimens are disregarded. In the second series of measurements (second batch), the bonded prisms with cover glasses also perform better, while the specimens with the drip edge perform worst by means of ACN and SD.

It can be seen that the coated prisms of S4 and S6 have a very high SD. The SDs for S4 range from 9.9–18.5. The SD for S6 is 17.5. This is due to the fact that the prisms are painted by hand and differ on the basis of the coatings. Furthermore, the 90° variant exhibits higher reflection signal than the variants bonded with cover glasses. This can be caused by surface tension of the lacquer leading to lens shaped surfaces that center the redirected light on the sensor of the photodiode. However, an influence of the angle cannot be clearly found here. The prisms with the bonded cover glasses (S5, as seen in Figure 4B) have the smallest SD which ranges from 1.6 to 3.6. Again, the 45° variant performs worse ACN‐wise. The specimen with the drip edge also has one of the highest standard deviations of all specimens, which is again most likely due to the uneven coatings. The designed drip edge though does not produce any improvement and probably favors the emergence of lens effects that direct the light with low degree of reproducibility. To summarize, the results of the experiments, for reproducible prisms with consistent quality, the surfaces should be bonded with cover glasses and in cases of impossibility, at least the surface should be coated with a transparent lacquer. On the base of the recent data, in cases of 45° orientation of coated prisms the SD of printed replicates is lower compared to 0° or 90° orientation.

### 3.2 Results OMW

#### 3.2.1 Characteristics

**FIGURE 5:**
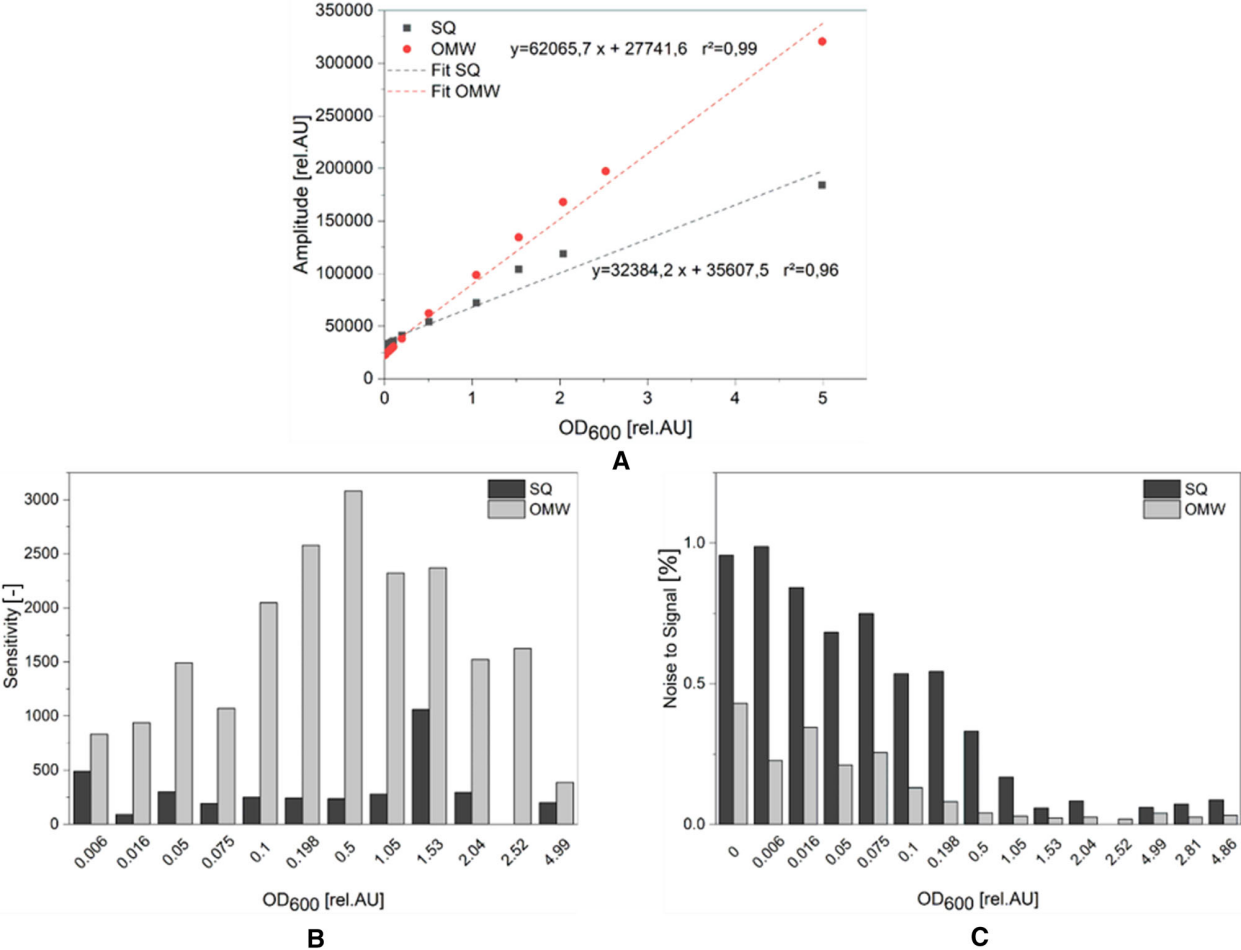
Calibration of the biomass sensor signal versus OD_600_ using a suspension of formazine. (A) Red circles represent the calibration carried out in the optically modified well (OMW). Grey squares represent the calibration of the Status Quo (SQ) well. Sensitivity (B) and Noise to Signal (NSR) (C) were calculated using Equations (5) and (6) of Section 3.2.1 respectively

First, calibrations were performed with the turbidity standard formazine in the OMW as well as in the SQ. The calibration range was selected so that the scattered light signal of formazine corresponds to an OD_600_ of a yeast suspension of 0.2 rel.AU. Formazine achieves a corresponding scattered light signal at an OD_600_ 0.018 rel.AU as a yeast suspension with an OD_600_ of 0.2 rel.AU. Figure 5A shows that the calibrations with the turbidity standard formazine lead to a correlation over the entire calibration range for both the SQ and the OMW. The linear fit for the OMW reaches a regression coefficient of determination of 0.99, slightly higher than that of the SQ with 0.96. However, the measurements with the OMW result in significantly higher amplitude signals at the same OD than the measurements with the SQ. Sensitivity was calculated using Equation (5)

(5) $$\mathrm{Sensitivity}\;\left[-\right]\;=\;\frac{\Delta \;Amplitude}{\Delta \;OD_{600}}\cdot \frac{1}{\sigma }$$

which results can be seen in Figure 5B. The sensitivity is a dimensionless value and reflects how much the value of the output variable (amplitude of the SFR vario) changes in relation to a change in the input variable (OD_600_). Δ is the difference of the mean values of two consecutive calibration points. σ is the calculated standard deviation of the measured values. For each calibration point, 10 measurement points were recorded (one measurement point every 30 s) and the noise to signal ratio (NSR) was calculated with Equation (6):

(6) $$\text{Noise}\;\text{to}\;\text{Signal}\;\text{ratio}\;\left[\%\right]=\frac{\sigma }{\bar{x}}\cdot 100$$

The NSR describes the variance within the calibration points where $\bar{x}$ is mean value of the measurement points for each calibration point. σ is the calculated standard deviation of the measured values. Figure 5C shows that for both the OMW and SQ, the NSR is significantly higher in the lower OD‐range and decreases with increasing OD. However, the measurements with the OMW show up to two times lower noise than those of the SQ. This shows that a significant reduction of the NSR can be achieved by the in‐plane scattered light measurement, since there is much less interfacial reflection compared to the SQ method.

**FIGURE 6:**
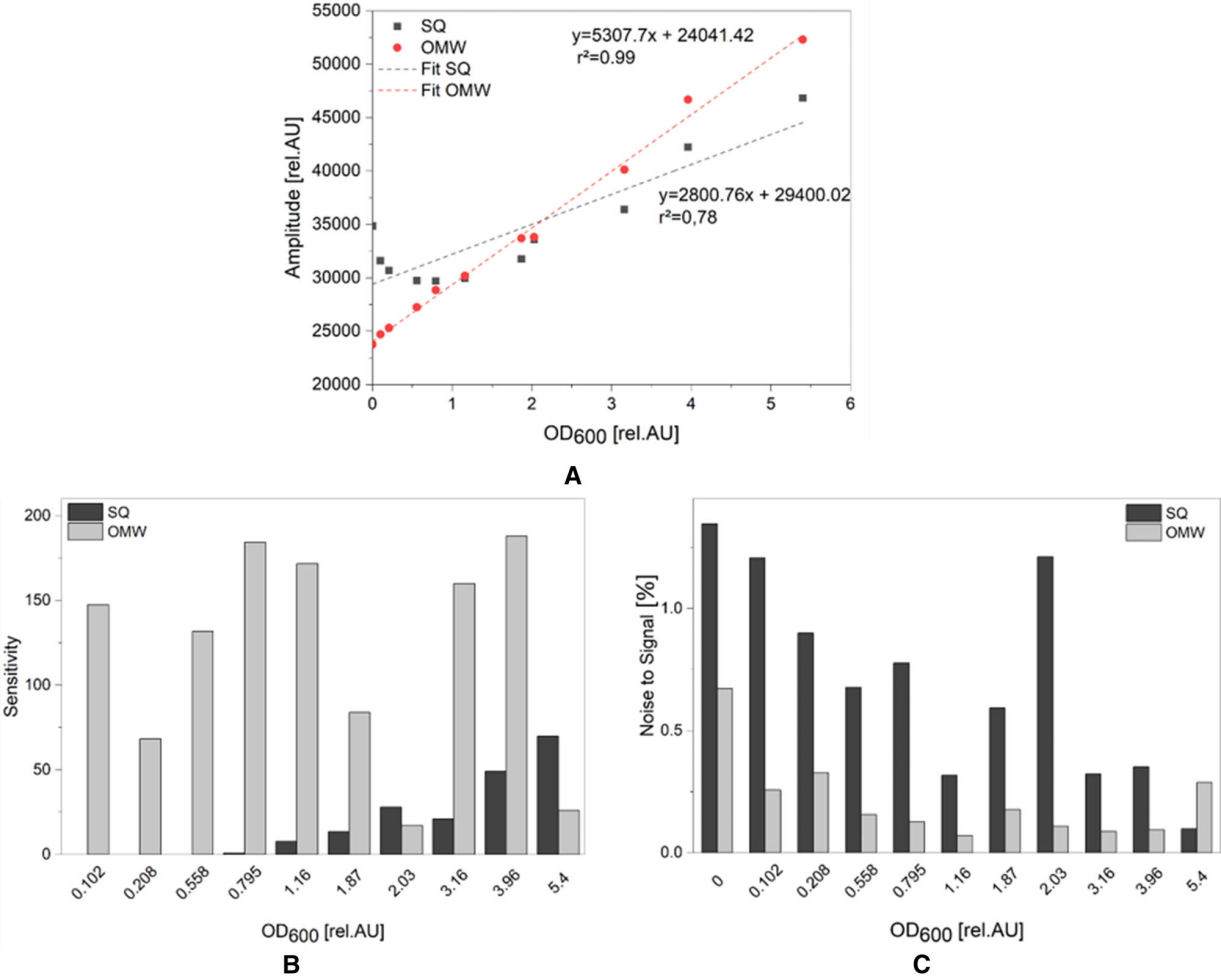
Calibration of the biomass sensor signal versus OD_600_ using a cell suspension of S. cerevisiae. (A) Red circles represent the calibration carried out in the optically modified well (OMW). Grey squares represent the calibration of the Status Quo (SQ) well. Sensitivity (B) and Noise to Signal (NSR) (C) were calculated using Equations (5) and (6) of Section 3.2.1 respectively

Since the OMW is intended to be used for cultivation of various organisms, a cell suspension of *S. cerevisiae* was applied for calibration as well. As can be seen in Figure 6A, the calibration graph of the SQ is clearly different in the case of using a cell suspension in comparison to formazine. Preceding research works showed that there is a high influence of the cell morphology on the scattered light signal [11]. Therefore, it can be assumed that the difference in the amplitude signal, under consideration of the same offline OD determination method, is due to the different scattered light behavior of formazine and an *S. cerevisiae* suspension per particle concentration unit. However, calibration with formazine suspension is adequate to reveal the differences in sensitivity of both optical setups (SQ and OMW).

This also leads to the conclusion that a valid prediction of calibration range and the limit of detection can only be stated using the very cell type which is used in calibrations later on as can be seen in Figure 6.

The calibration graph of the SQ shows no linear behavior at low OD (0–1.2 rel. AU). Although the dilution series is in ascending order, the amplitude signal values of the biomass sensor decrease in the range of OD_600_ 0–1.16 rel.AU. For the SQ this means at a OD_600_ = 1.2 rel.AU is the limit where the OD_600_ can be reliably measured which is near to the lower limit of the measuring range (OD_600_ 1–80) given by the manufacturer of the SFR vario [21]. Figure 6A clearly displays that a linear correlation between sensor amplitude and offline determined OD_600_ over the total low OD‐range can be achieved using the OMW. Using the Equations (3) and (4) the LoD and LoQ are calculated with 0.10 rel.AU and 0.30 rel.AU, respectively. Therefore, decreasing the lower measuring range for the SFR vario to OD_600_ 0.1 for *S. cerevisiae*. Sensitivity is also at least three times higher compared to the SQ measurements (see Figure 6B). Using *S. cerevisiae* for calibration procedure results in a similar NSR than using formazine. Nevertheless, regarding SQ a drop below a NSR of 0.5% can be seen at OD > 0.2 rel.AU of formazine, while for *S. cerevisiae* the same is not visible before OD_600_ =  3.2 rel.AU, which underlines the aforementioned dependency of scattered light on the particle type. To further investigate the durability of the optics and measuring range of the newly optical system a full batch cultivation with *S. cerevisiae* was performed with the OMW for 24 h (Figure S1). No damage can be observed on any printed parts of the OMW. This further shows how 3D‐printed cultivation vessels can be used in harsh conditions and also shows the robustness of the lacquer used. Also, the OMW can be used for measuring values up to but not limiting to OD_600_ = 30 rel.AU.

#### 3.2.2 Relocation effect and influence of batch to batch variation

The well is mounted on the SFR vario by three screws, which results in a certain tolerance for the location. Therefore, it should be investigated to what extent a variation in mounting position affects the scattered light signal. For this purpose, the same well was mounted and dismounted 10 times and the scattered light signal was determined in each case. The calculated standard deviation of the scattered light signal is 0.9%. Thus, the influence of the placement on the measurement signal is negligible.

**FIGURE 7:**
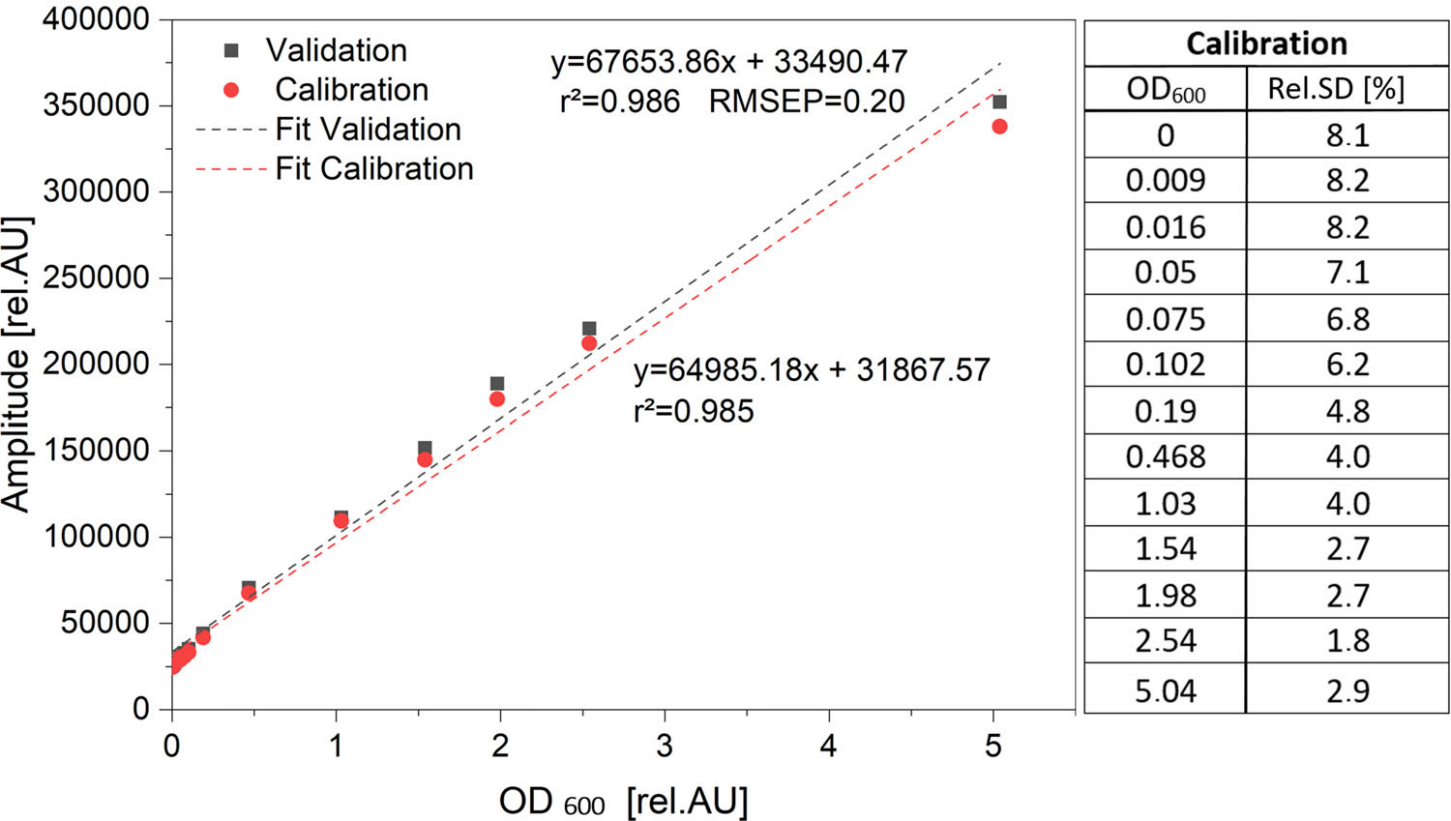
Calibration of the biomass sensor signal versus OD_600_ using turbidity standard formazine. Red circles represent the calibration of three experiments in three different wells (corresponding relative SDs in the chart next to it). Grey squares represent one additional validation experiment

In addition to the relocation effect, batch dependency plays a major role in single‐use (SU) applications. To investigate the batch dependence, three wells were printed. Subsequently, a calibration with the turbidity standard formazine was performed in each of these wells. Red circles in Figure 7 represent the calibration of the three calibration experiments. The relative standard deviation decreases with increase of the OD. In the OD_600_‐range of 0–5.04 rel.AU, the relative standard deviation decreases from 8.1 to 2.9%. This shows that variations in printing or processing lead to deviations especially in the lower OD‐range.

Since three different 3D‐printed OMWs were used, the RMSEP also contains the batch variance of reprints. The calculated RMSEP is 0.2, so the accuracy for the OMW is 20% for OD_600  _=  1, which is comparable to the accuracy of commercial biomass sensor, which have an accuracy of about 15% [22]. Since the accuracy always depends on the microorganisms cultivated, this result should only be used for a first impression of the accuracy. Further investigations could be a part of future works.

By transferring the experimental results of Section 3.1 to the OMW it was confirmed that most relevant parameters for biomass sensor accuracy are linked to the deviation caused by variations between print batches. Uneven surfaces are caused by layer management in the slicer software, printing angles, post processing and modifications like coatings and bonding of glass covers.

While the relative SD for prism S5_45°_3GCS is 2.9% the average SD of OMW replicates is 5.25%. Therefore, a slightly higher deviation of printed OMW replicates was found using LED light at a dominance beam angle of 50° (SFR vario + OMW) compared to single prism replicates evaluated by collimated laser light. Consequently, the laser method and optical bench for the reflective optics evaluation did not suggest a biased performance for good optics, but rather a realistic assessment for achievable characteristics.

## 4 DISCUSSION

The aim of this work was to develop a simple and reliable method how to purposefully print and post modify prisms to evaluate whether functional light guiding elements can be integrated into 3D‐printed SU minibioreactor vessels. It was shown that the integration of prisms into the well and the resulting change in the light guidance of the scattered light measurements from vertical to horizontal leads to higher linearity and sensitivity whereby the NSR can be significantly reduced. The linearity of turbidity measurement could be significantly improved by integrating the 3D‐printed prisms into the vessel, especially in the low OD‐range, which could enable for the first time the application of SFR vario for mammalian cell cultures with low cell densities. From product development perspective 3D‐printing proved to be an effective and cheap option for evaluation of complex injection mold prototypes, considering that injection molds itself are expensive and changes in their design are time consuming. Moreover, it could be shown that print variations of the OMW are within an acceptable range for parallel screening applications using SU vessels, bioreactors respectively.

It is pointed out that some material parameters like absorbance are neglected but could be improved by annealing processes, for example, tempering [23]. Not only can the mechanical properties be improved but optical properties as well, thus reaching higher luminous efficacy. Future improvements can also be made to the optical bench. A photodiode with a larger photosensitive area would lead to more accurate results. In addition, it is still necessary to investigate how to more uniformly and reproducibly coat the prisms with the lacquer, since bonding of glass is not always possible in general. Although the results of the measurements were promising, the reproducibility of these coating methods was not. Therefore, automatic spray or vapor deposition coating methods would be a worthwhile step ahead for in bigger part numbers to be evaluated.

There is a general interest in 3D‐printed optics for future applications and how to edit them to reach for certain functionalities [24, 25]. Within the last years the market for 3D‐printers that can print optical parts of very high transparency expanded tremendously.

At the moment, specialized printers are used and constantly improved in ophthalmology and the eyeware industry [26].

There are many applications of 3D‐printing of bioreactors [27]. Also, 3D‐printing is used in lab on a chip (LOC) devices which use transparent windows for analytical procedures [28]. However, currently there is no commercial system containing cultivation vessels where 3D‐printing is used to combine complex optics and vessel in one unibody design.

Since this research was successful and gives information about crucial bottlenecks and optimization parameters in 3D‐printing, it should be determined whether integrated optics can also be used to examine other parameters in small cultivation vessels or flow through cells.

## CONFLICT OF INTEREST

The authors declare no conflict of interest.

## Supporting information

SUPPORTING INFORMATION

SUPPORTING INFORMATION

## Data Availability

The data that support the findings of this study are available from the corresponding author upon reasonable request.
