# Supplementary material for: Study on the development and integration of 3D‐printed optics in small‐scale productions of single‐use cultivation vessels
Source: Eng Life Sci. 2022 Mar 18;22(6):440–52. doi: 10.1002/elsc.202100131 (PMC9162927; doi:10.1002/elsc.202100131)
Supplement: Supplementary file 1 — SUPPORTING INFORMATION [file ELSC-22-440-s002.pdf]

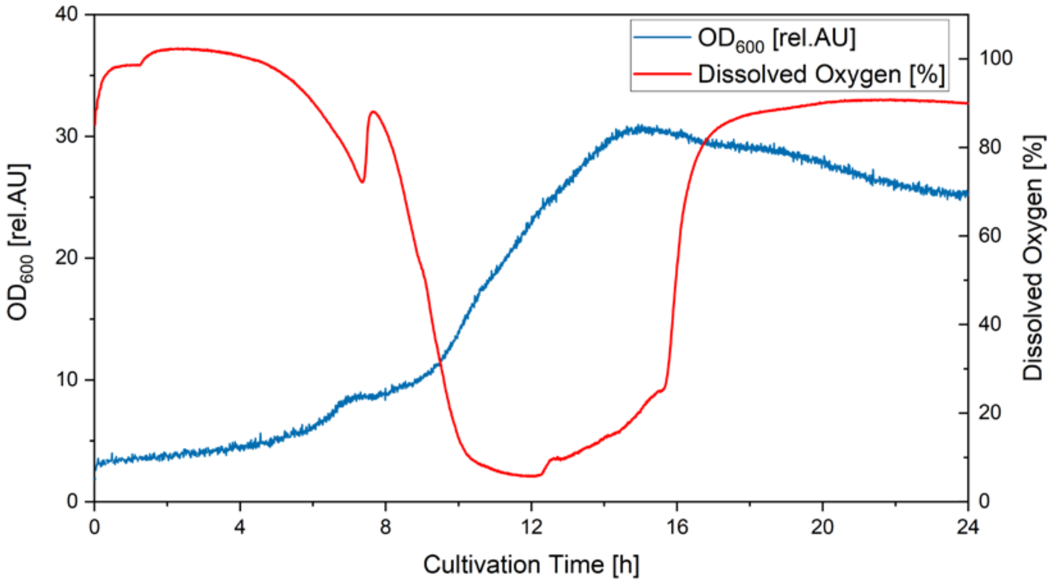

Figure S1. Growth and oxygen values of a batch cultivation of *S. cerevisiae* with the optically modified well (OMW) using the shake flask reader (SFR) vario. Cultivation was achieved with a YPD media (10 g/L yeast extract, 20 g/L peptone, 10 g/L glucose). Oxygen was measured with the SFR vario using a sensor spot SP-PSt3-YAU-D5 (PreSens Precision Sensing GmbH). An orbital shaker with 25 mm shaking diameter (Solaris™ 2000, ThermoFisher Scientific, Waltham, USA) was used. Cultivation was carried out at 30° C.
