# Supplementary figures and images for: Study on the development and integration of 3D‐printed optics in small‐scale productions of single‐use cultivation vessels
Source: Eng Life Sci. 2022 Mar 18;22(6):440–52. doi: 10.1002/elsc.202100131 (PMC9162927; doi:10.1002/elsc.202100131)

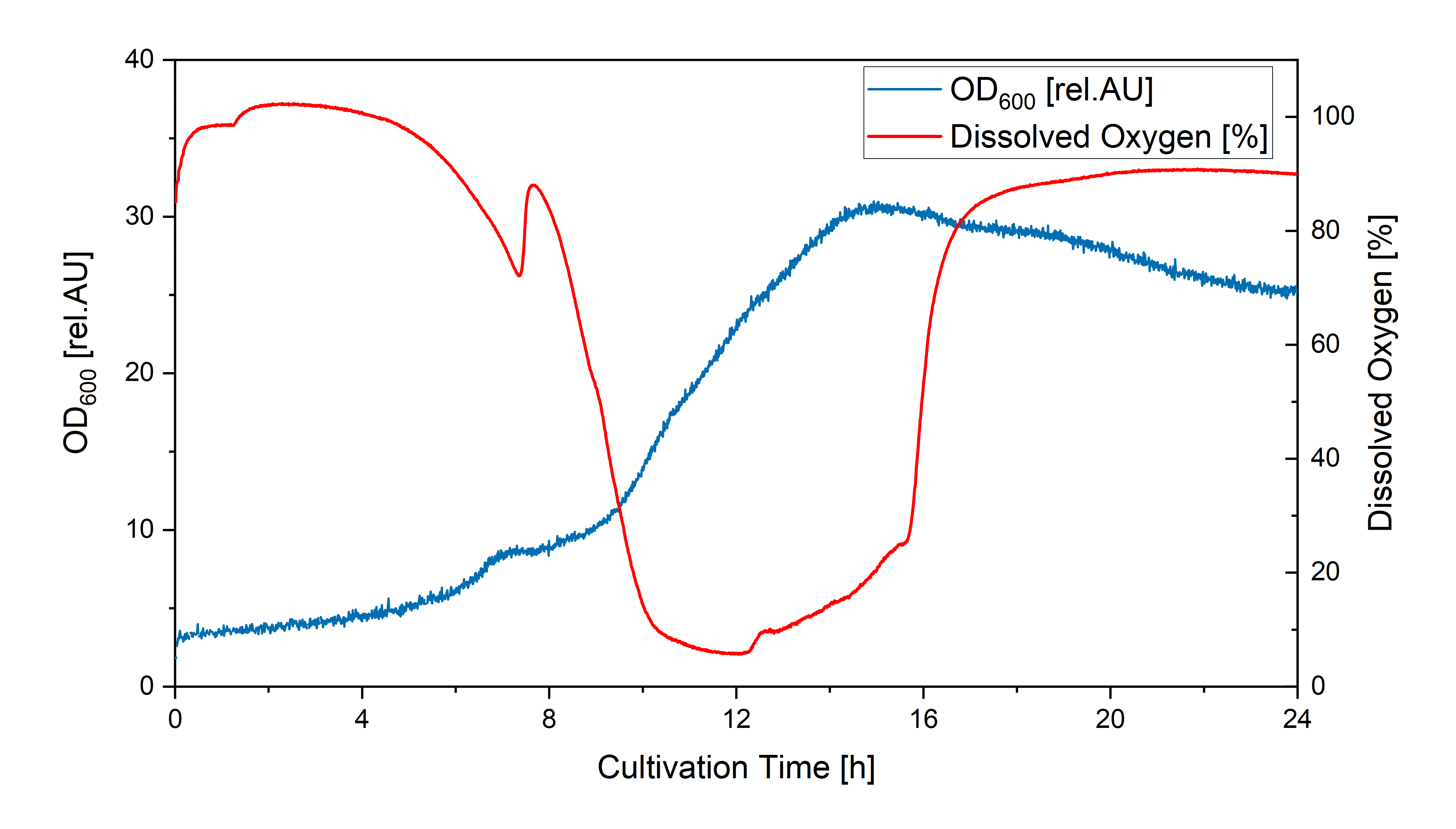

Supplement: Supplementary file 2 — SUPPORTING INFORMATION [file ELSC-22-440-s001.png]
